# Noise Suppressed Image Reconstruction for Quanta Image Sensors Based on Transformer Neural Networks

**DOI:** 10.3390/jimaging11050160

**Published:** 2025-05-17

**Authors:** Guanjie Wang, Zhiyuan Gao

**Affiliations:** School of Microelectronics, Tianjin University, 92 Weijin Road, Tianjin 300072, China

**Keywords:** quanta image sensors, photon counting, image reconstruction, image denoising

## Abstract

The photon detection capability of quanta image sensors make them an optimal choice for low-light imaging. To address Possion noise in QIS reconstruction caused by spatio-temporal oversampling characteristic, a deep learning-based noise suppression reconstruction method is proposed in this paper. The proposed neural network integrates convolutional neural networks and Transformers. Its architecture combines the Anscombe transformation with serial and parallel modules to enhance denoising performance and adaptability across various scenarios. Experimental results demonstrate that the proposed method effectively suppresses noise in QIS image reconstruction. Compared with representative methods such as TD-BM3D, QIS-Net and DPIR, our approach achieves up to 1.2 dB improvement in PSNR, demonstrating superior reconstruction quality.

## 1. Introduction

With the continuous advancement of Moore’s Law, the resolution of conventional complementary metal-oxide-semiconductor image sensors (CIS) has steadily increased, leading to a reduction in pixel size. However, at the deep sub-diffraction-limit (deep-SDL) scale, smaller pixels result in decreased full well capacity (FWC), which have a negative impact on signal-to-noise ratio (SNR) and limits the dynamic range of the sensors [1]. To address these limitations, the concept of the digital film sensor (DFS) was proposed in 2005 [2], and was formally termed the quanta image sensor (QIS) in 2011 [3]. Recent advances [4] reveal that the latest QIS prototype has achieved a read noise below 0.2 e- r.m.s at room temperature, with a pixel resolution of 16.7 million and a pixel pitch of 1.1 μm. This state-of-the-art sensor is expected to incorporate smaller pixel pitches, higher frame rates, lower read noise, and reduced dark current while maintaining high performance at room temperature and production costs comparable to traditional CIS [5]. These features make QIS highly suitable for low-light imaging applications. Numerous studies in computer vision have demonstrated the effectiveness of the sensor in addressing low-light scenarios [6,7,8,9].

**Figure 1:**
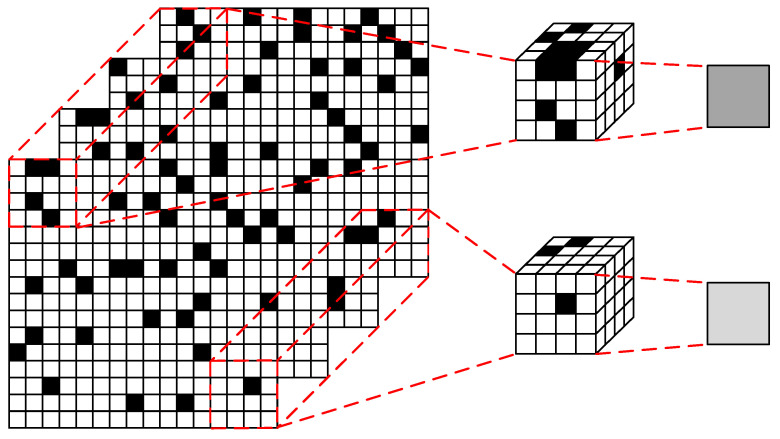
Concept of spatio-temporal oversampling.

The QIS is built on an array of ultra-small photodetectors referred to as ‘jots’ [10], each of which is quantized using an analog-to-digital converter (ADC). A distinguishing feature of QIS is its ability to perform spatio-temporal oversampling through high-frequency sampling. In this context, the oversampling unit, known as a ‘cubicle’, is composed of 3D arrays of ‘jots’. The main purpose of the spatio-temporal oversampling process is to suppress Poisson noise induced by photon incident events, thereby improving image quality [11]. After oversampling, a reconstruction process is carried out to convert the data in the ‘cubicle’ into reconstructed pixel values. The concept of spatio-temporal oversampling is illustrated in Figure 1.

The analysis of noise and bit error rates in QIS was first introduced in [12,13], followed by detailed mathematical modeling and SNR formulations [14]. One of QIS’s key advantages is its ability to programmatically adjust spatial and temporal oversampling rates for high SNR image reconstruction [10]. Therefore, robust reconstruction algorithms are essential for achieving high-quality imaging. Early research framed image reconstruction as a photon number estimation challenge. Yang et al. [15] proposed a Maximum Likelihood Estimation (MLE)-based algorithm for this purpose and Chan et al. [16] proposed a Maximum A Posteriori (MAP) estimation based on the Alternating Direction Method of Multipliers (ADMM). In addition to photon number estimation methods, a non-iterative reconstruction method based on Block-Matching and 3D Filtering (BM3D) denoiser [17] and a deep learning reconstruction method based on convolutional neural networks (CNN) [18] have also been proposed.

The photon detection capability makes QIS an optimal choice for imaging in low-light conditions. However, the low SNR bit-planes pose challenges to image reconstruction, thus the appropriate noise suppression method can improve the quality of reconstructed images. The photon number estimation methods [15,16] suppress noise based on imaging principles. However, the estimated values of these methods significantly deviate from the true values and the mathematical expressions become very complex and difficult to solve as the bit depth increases. Additionally, the non-iterative BM3D method [17] utilizes complex frequency domain filtering algorithms to achieve better denoising results compared to photon number estimation methods. However, the time complexity of this method is high and the performance of the BM3D denoiser has been proven to be lower compared to the denoisers of the neural network [19]. The QISNet reconstruction method [18], based on CNN, has been proposed for noise suppression in QIS image reconstruction. Nevertheless, with the continued development of denoising technology, more complex neural network denoisers for QIS reconstruction need to be considered.

In recent years, various filtering techniques have been developed to improve image denoising performance. Traditional methods, such as Gaussian smoothing, median filtering, and particularly Wiener filtering, have laid the groundwork for noise suppression by modeling local statistics and frequency domain characteristics. A comprehensive review of these approaches can be found in [20]. Building on these classical methods, filtering approaches based on doubly stochastic models have demonstrated strong potential in handling signal-dependent noise through adaptive probabilistic frameworks. For example, ref. [21] proposed an image restoration method using a doubly stochastic model, while [22] introduced a noise suppression approach that considers the two-layer stochastic nature of image acquisition. These methods provide important theoretical foundations for filtering under complex noise characteristics. With the rapid development of data-driven techniques, deep learning-based methods have also shown impressive results in recent years. These methods are capable of learning powerful priors directly from data and have demonstrated state-of-the-art performance in a wide range of denoising tasks. A recent survey by [23] provides a comprehensive overview of the progress in this area.

The unique hardware characteristics of QIS inherently provide superior capability for low-light imaging. By leveraging high-speed frame acquisition and high temporal oversampling rate, QIS can achieve imaging even in extremely low-light conditions. However, this comes at the cost of longer imaging time and significantly increased data volume. More specifically, we contribute to the literature of QIS image reconstruction as follows:

- This study propose a reconstruction method that suppress photon shot noise during the QIS imaging process under a practical and acceptable temporal oversampling rate based on deep neural network instead of traditional denoising.
- The proposed neural network framework integrates CNN and Transformer to improve denoising performance. A hybrid structure combining serial and parallel is introduced in the network framework to enhance the strength and robustness of denoising. The serial module deeply explores the key information in image denoising, while the parallel module widely explores more relevant and complementary information between pixels from different angles, thereby enhancing the adaptability of the QIS-SPFT denoiser to complex scenes. In the network, a variance-stabilizing transformation is used to convert the high Poisson noise in QIS into Gaussian noise to enhance the performance as well.

The remainder of the paper is organized as follows. Section 3 introduces the background of QIS imaging. Section 4 provides a detailed theoretical analysis of the proposed image reconstruction method. Section 5 presents and compares experimental results. Finally, Section 6 concludes the paper.

## 2. Background

**Figure 2:**
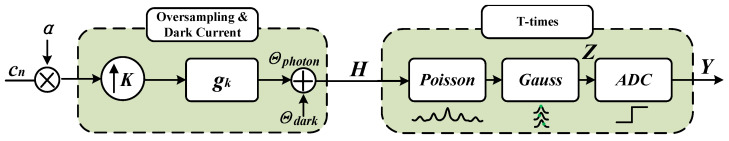
The imaging model of the QIS.

In this section, the noise composition of the QIS is analyzed and the mathematical model for imaging is provided. The formulation of the QIS imaging model is fundamental to QIS image reconstruction. Based on the QIS imaging principle, a QIS imaging model is established, as shown in Figure 2.

Consider a light wave with intensity I(x,y,t) incident on a sensor with duty cycle *T* and integration time Δ. The mean number of photons, denoted as θ, received by a ‘jot’ with an area *A* during the integration time is expressed as follows:

(1) $$\theta =\underset{(x,y)\in A}{\;\int \int \;}\int_{T}^{T+\Delta }I\left(x,y,t\right)dxdydt,$$

The probability of photon number *Y* the ‘jot’ which has a small size and a short time interval get satisfy the Poisson distribution:

(2) $$P\left(Y=y\right)=\frac{e^{-\theta }\theta^{y}}{y!},$$

Dark current, generated by pixels in the absence of light, leads to the production of dark electrons, affecting the accuracy of photon counting. These electrons also follow a Poisson distribution.

When considering spatio-temporal oversampling, the image information containing *N* light intensity $c_{i}$ is reconstructed from M oversampled ‘jots’ $\theta_{i}$. The spatio-temporal oversampling rate is represented as K=M/N.

If we use matrix-vector notation, the Poisson distribution of photons is represented as:

(3) $$\Theta_{\mathrm{photon}}=\mathrm{Gc},$$

where $c={\left[c_{0},\ldots ,c_{N-1}\right]}^{T}$ denotes the reconstructed light intensity, $\Theta_{\mathrm{photon}}={\left[\theta_{0},\ldots ,\theta_{M-1}\right]}^{T}$ denotes the light intensity sampled at the *M* jots, and the matrix $G\in R^{M\times N}$ is a matrix representing the upsampling and the lowpass filter $g_{k}$. The lowpass filter $g_{k}$ can be assumed as a box-car. Now, total photons can be described as:

(4) $$\Theta_{\mathrm{tot}}=\Theta_{\mathrm{photon}}+\Theta_{\mathrm{dark}},$$

(5) $$P\left({\left[Y\right]}_{l}=y\right)=\frac{{\left[\gamma \cdot \Theta_{\mathrm{tot}}\right]}_{l}^{y}e^{-{\left[\gamma \cdot \Theta_{\mathrm{tot}}\right]}_{l}}}{y!},$$

where l=1,…,N, γ is the sensor gain, $\Theta_{\mathrm{tot}}\in R^{N}$ is the mean number of electrons physically and Y is the electrons really sensed by pixels.

Once the electrons are sensed by the pixels, they undergo readout, which the analog circuit generates additive Gaussian noise, denoted as, $\eta_{read}∼N\left(0,\sigma_{read}^{2}\right)$. Thus, the real number of electrons $Z\in R^{N}$ follows a Gaussian-Poisson distribution:

(6) $$\begin{array}{c} P\left({\left[Z\right]}_{l}=z\right)=\sum_{y=0}^{\infty }(\frac{{\left[\gamma \cdot \Theta_{\mathrm{tot}}\right]}_{l}^{y}e^{-{\left[\gamma \cdot \Theta_{\mathrm{tot}}\right]}_{l}}}{y!}\cdot \frac{1}{\sqrt{2\pi \sigma_{read}^{2}}}e^{-\frac{{\left(z-y\right)}^{2}}{2\sigma_{read}^{2}}}), \end{array}$$

After readout, electrons are converted into digital codes by the ADC. Due to the BER [12] caused by mismatched ADC transition points, there exists difference between ‘jots’ [24]. In conclusion, the quantization process is modeled as:

(7) $$Q=\left\{ADC(Gauss-Poiss(\gamma \cdot \Theta_{\mathrm{tot}};\sigma_{read})+O)\right\},$$

where $O\in R^{N\times N}$ represents ADC transition points mismatch.

## 3. Method Description

### 3.1. Architecture of the Proposed QIS Reconstruction Method

This paper introduces a neural network architecture named QIS-SPFT (QIS Serial-Parallel Fusion Transformer), which integrates both serial and parallel networks within Transformer frameworks to suppress Gaussian-Poisson noise in QIS reconstruction.

The QIS-SPFT method capitalizes on the interaction of diverse structural information, obtained through attention mechanisms in both breadth and depth from various serial and parallel networks, to extract salient features for improved reconstruction of clean images. Its superior denoising performance is achieved through the Serial Module (SM) and Parallel Module (PM). The SM utilizes linear and nonlinear components to thoroughly search for essential information in image denoising. To gather more complementary information from different perspectives, the PM extensively explores interactions and cross-features between pixels obtained from two mixed networks, Subnet1 and Subnet2, thereby enhancing the network’s adaptability to complex noise.

**Figure 3:**
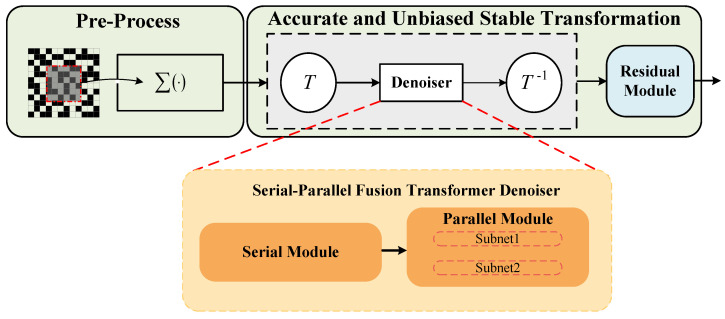
Architecture of the proposed QIS reconstruction method.

The denoising framework QIS-SPFT use the Anscombe transformation, which makes the noise in jot-summation images closer to Gaussian distribution with constant variance. This variance-stabilizing technique facilitates the suppression of Poisson noise in QIS images. In this study, the neural network is incorporated as a denoiser within the QIS image reconstruction, and the neural network needs to learn to suppress the noise after Anscombe transformation. The architecture of the proposed QIS reconstruction method is shown in Figure 3.

Within this network framework, a deep denoising network module is employed to thoroughly capture the structural information of images after variance stabilization. This module primarily comprises convolutional layers, activation functions, and Transformers. The convolutional layers convert the noisy images into linear features, the nonlinear activation functions extract richer features, while the Transformers discover the relationship between different image blocks, thus enhancing denoising performance in complex scenarios. Additionally, the framework integrates a robustness-enhancement network module, where two parallel sub-networks interact to extract complementary features from diverse perspectives, thereby improving the model’s robustness and adaptability to various scenarios. To further enhance denoising performance, Transformers are embedded in both serial and parallel modules to extract more prominent features and effectively filter out noise. This network can be represented as:

(8) $$Resid^{-1}\left(I_{C}\right)=RM\left(PM\left(SM\left(I_{N}\right)\right)\right),$$

where $I_{N}$ represents the pre-reconstructed image, and SM, PM, and RM are defined as functions of the Serial Module, Parallel Module, and Residual Module. $I_{C}$ is the clean image processed by the network. Further details about each module will be described in next subsection.

To objectively assess the denoising performance of the proposed QIS-SPFT, the Mean Squared Error (MSE) is employed as the loss function for training the network parameters. Specifically, MSE is utilized to train the denoiser with paired training samples $I_{C}^{i},I_{N}^{i}$(1≤i≤n), where $I_{C}^{i}$ and $I_{N}^{i}$ denote the i-th clean and noisy images in the training dataset, respectively, and *n* represents the total number of training samples. This training process can be mathematically expressed as:

(9) $$l\left(\theta \right)=\frac{1}{2n}\sum_{i=1}^{n}{\left|\mathrm{QIS}\text{-}\mathrm{SPFT}\left(I_{N}^{i}\right)-I_{C}^{i}\right|}^{2},$$

where, *l* represents the loss function, and θ denotes the parameters required for training QIS-SPFT.

### 3.2. Detail of the QIS-SPFT Denoiser

**Figure 4:**
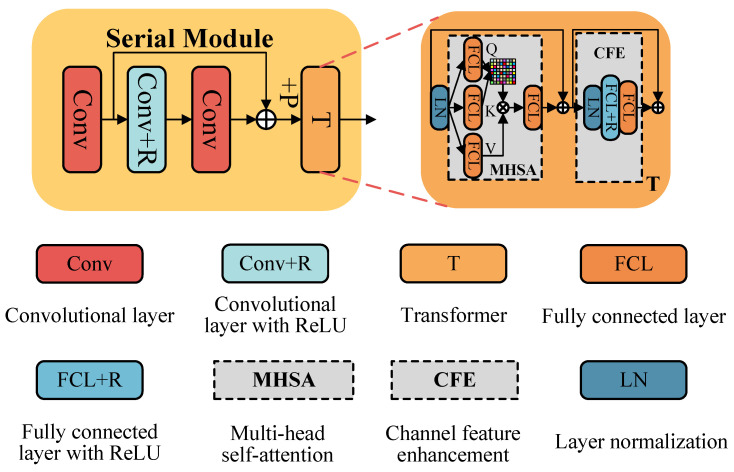
Detail of the serial module.

The SM is utilized to extract structural information from quanta images that contain complex noise components. Its superior denoising performance is attributed to its serial architecture, which is primarily composed of three components: convolutional layers, activation functions, and Transformers. The first and third convolutional layers employ Conv. Given that this study focuses on grayscale noisy images directly generated by light intensity, the input and output channels of the first convolutional layer are set to 1 and 64, respectively. The second convolutional layer, Conv+R, integrates linear and nonlinear components to extract richer features. In this context, Conv+R is defined as the combination of a convolutional layer, denoted as Conv, and an activation function, specifically Relu. The convolutional layer Conv extracts linear features, whereas the Relu function acts as a piecewise function, transforming linear information into nonlinear features. The Transformer structure is employed to explore relationships between different patches and to dynamically learn weights for various inputs, enabling adaptive noise reduction across diverse areas in the image. The architecture of SM is shown in Figure 4 and the output of SM is mathematically represented by Equation (10).

(10) $$\begin{array}{cc} O_{SM} & =SM(I_{N})\\ \; & =T(C\left(C\left(R\left(C\left(I_{N}\right)\right)\right)+C\left(I_{N}\right)\right), \end{array}$$

In this network, the Transformer architecture is implemented through an encoder that incorporates Multi-Head Self-Attention (MHSA) and Channel Feature Enhancement (CFE) mechanisms. These components are mathematically represented by Equation (11).

(11) $$\begin{array}{cc} T(I_{T}) & =CFE\left(O_{MHSA}+I_{T}\right)+I_{CFE}\\ \; & =FCL\left(FCL\left(R\left(LN\left(O_{MHSA}+I_{T}\right)\right)\right)\right)+I_{CFE}, \end{array}$$

where, FCL denotes the fully connected layer, LN represents layer normalization, $I_{T}$ is the Transformer input, $O_{MHSA}$ is the MHSA output, and $I_{CFE}$ is the CFE input. The MHSA mechanism extracts global contextual information to enhance significant features for image denoising. It employs normalization layers to standardize the feature distribution, which are then fed into three parallel branches, each comprising a fully connected layer. The outputs of these branches are designated as *Q*, *K*, and *V*. The features are integrated via self-attention, further refined through an FCL, and then used as the MHSA output. Residual operations merge features from Transformer inputs and MHSA outputs into the CFE. This process is described by Equation (12):

(12) $$\begin{array}{cc} O_{SM} & =T(O_{IN\_T})\\ \; & =CFE\left(MHSA\left(O_{IN\_T}\right)+O_{IN\_T}\right)+O_{IN\_CFE}\\ \; & =CFE(FCL(\mathrm{softmax}\\ \; & \left(\frac{FCL\left(LN\left(O_{IN\_T}\right)\right)\times FCL{\left(LN\left(O_{IN\_T}\right)\right)}^{T}}{d}\right)\\ \; & \times FCL\left(LN\left(O_{IN\_T}\right)\right))+O_{IN\_T})+O_{IN\_CFE}\\ \; & =CFE\left(FCL\left(\mathrm{softmax}\left(\frac{Q\times K^{T}}{d}\right)\times V\right)+O_{IN\_T}\right)+O_{IN\_CFE}\\ \; & =CFE\left(O_{MHSA}+O_{IN\_T}\right)+O_{IN\_CFE}, \end{array}$$

The MHSA mechanism is illustrated in Equation (12), which efficiently captures global structural information. Concurrently, normalization layers and fully connected layers are employed to standardize features, thereby improving denoising performance. The output of the MHSA is then further processed through a series of fully connected layers and normalization steps within the CFE mechanism, where it is combined with the original inputs to achieve a more effective representation of clean image information.

**Figure 5:**
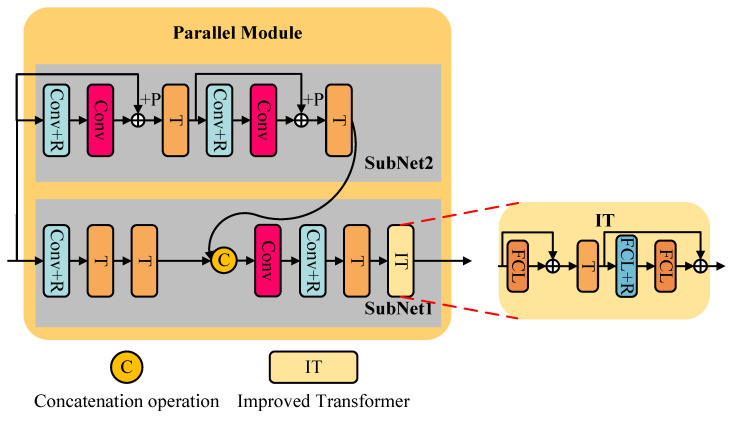
Detail of the parallel module.

As shown in Figure 5, the parallel module consists of two interacting parallel subnetworks that capture complementary features at different levels. To enhance the effectiveness of deep learning, a residual learning mechanism is employed in the subnetworks, where the residual between the input to the convolutional layer and the output after the convolutional layer is computed and used as the input to the Transformer. This process can be expressed by Equation (13).

(13) $$\begin{array}{cc} O_{PM} & =PM(O_{SM})\\ \; & =O_{Subnet1}\\ \; & =Subnet1(O_{SM},O_{Subnet2}), \end{array}$$

where $O_{PM}$ is the output of the parallel module, and $O_{Subnet2}$ is the output of subnetwork 2. In the parallel module, subnetwork 2 can be expressed by Equation (14).

(14) $$\begin{array}{cc} O_{Subnet2} & =Subnet2(O_{SM})\\ \; & =T(C\left(CR\left(T\left(C\left(CR\left(O_{SM}\right)\right)+O_{SM}\right)\right)\right))\\ \; & +\;T(C\left(CR\left(O_{SM}\right)\right)+O_{SM})), \end{array}$$

Subnetwork 1 can be expressed by Equation (15).

(15) $$\begin{array}{cc} O_{Subnet1} & =Subnet1(O_{SM},O_{Subnet2})\\ \; & =IT(T(CR(C(Concat(T(T(\\ \; & CR\left(O_{SM}\right))),O_{Subnet2}))))), \end{array}$$

The SM deeply searches for key information in image denoising, while the PM extensively explores more relevant and complementary information between pixels from different perspectives to enhance the adaptability of the QIS-SPFT denoiser to complex scenes. Its effectiveness is achieved through the interaction of two heterogeneous networks, namely subnetwork 1 and subnetwork 2, at multiple feature levels. These two subnetworks interact and acquire complementary features from different perspectives to enhance the robustness of the obtained denoising model. The Conv+R component is used to extract nonlinear information, while the Conv component is used to extract linear information. The *T* component functions between Conv and Conv+R to extract salient information. Furthermore, to augment the denoising network’s capability, the input of Conv+R and the output of Conv are integrated using a residual learning operation.

In the parallel module (PM), two robustness enhancement operations are employed. The first operation aims to improve the robustness of the features obtained from the denoiser by introducing an interaction mechanism between subnetwork 1 and subnetwork 2, namely the merging operation Concat. The Concat operation includes depthwise separable convolution layers, enabling information sharing and mutual influence between the two subnetworks, thereby enhancing the module’s robustness against noise. Here, robustness refers to the module’s ability to maintain effective denoising performance when facing different types of noise or complex noise conditions. The second operation, following the information interaction between different subnetworks, incorporates an improved Transformer component, IT, to eliminate interference information from previous interactions. The IT component consists of the stacks FCL, *T*, FCL+R, and FCL. Additionally, to enhance information acquisition, residual learning operations are conducted between the input of IT and the output of FCL, as well as between the outputs of *T* and FCL.

The residual module (RM) consists of a convolutional layer and a residual learning operation, aiming to construct a clean image by performing residual learning between the original noisy image and the predicted image in QIS-SPFT. The original image in this module is the noisy image before the Anscombe transformation, while the predicted image is the one obtained after the inverse Anscombe transformation. The convolution kernel size in this module is 3×3, with 64 input channels and 1 output channel. RM can be expressed by Equation (16).

(16) $$\begin{array}{cc} I_{c} & =RM(O_{PM})\\ \; & =I_{N}-C\left(O_{PM}\right). \end{array}$$

## 4. Experimental Results

In the following subsection, simulations and experimental validations were conducted for the QIS noise suppression method proposed in this paper. In this section, the effectiveness of the network architecture is first verified through ablation experiments, and then the proposed reconstruction method is evaluated across different datasets. All experiments were performed in a Python 3.11.4 environment on a computer equipped with an Intel 12400F/4.4 GHz 6-core CPU and 32 GB of memory and the neural network training was accelerated using an Nvidia Geforce RTX 3070Ti with CUDA 12.3.

The training dataset consisted of the following: the BSD dataset containing 432 natural images [25], the DIV2K dataset containing 800 natural images [26], the Flickr2K dataset containing 2650 natural images [27], and the WED dataset containing 4744 natural images [28]. This formed a synthetic noise training dataset with a total of 8626 natural images. To enrich the training dataset, each image was randomly cropped into 108 patches of size 48×48, resulting in 931,608 image patches for training the grayscale denoising model. The input images used for training were the $\frac{S_{n}}{L}$, which are summed three-dimensional data matrices under temporal oversampling rate *L*. During training, the temporal oversampling rate *T* was set to 16, the spatial oversampling rate *K* was set to 1, and $\sigma_{\mathrm{read}}$ was set to 0.2e-. The targets are the corresponding clean (ground-truth) images. For testing and evaluation, we adopt the standard benchmark datasets BSD68, Set12 [29], and Kodak24 [30].

The parameter settings for training the QIS-SPFT denoiser were as follows: batch size was set to 8, epochs were set to 24, and the initial learning rate was set to $1\times 10^{-4}$.

### 4.1. Network Analysis Experiments

To explore the rationality of the network structure settings in this study, experiments were conducted to compare the configurations with and without Anscombe transform, with and without the IT component, in a combinatorial manner. The test datasets were Set12 [29] and BSD68 [29], with Noise level 1 (*T* = 16, *K* = 4, $\sigma_{\mathrm{read}}$ = 0.2e-), Noise level 2 (*T* = 16, *K* = 4, $\sigma_{\mathrm{read}}$ = 0.4e-), Noise level 3 (*T* = 16, *K* = 2, $\sigma_{\mathrm{read}}$ = 0.2e-), Noise level 4 (*T* = 16, *K* = 1, $\sigma_{\mathrm{read}}$ = 0.4e-). This paper objectively evaluates the denoising effect using peak signal-to-noise ratio (PSNR), which can be expressed as:

(17) $$\mathrm{PSNR}=10\log_{10}\left[\frac{{\left(2^{n}-1\right)}^{2}}{MSE}\right]$$

where *n* is the bit-depth of the image, which is generally set to 8. MSE is the mean squared error between images.

We compute the average PSNR results of processed reconstructed images across multiple datasets characterized by differing levels of noise under various network architectures. The comparison result of PSNR across various network architectures is shown in Table 1.

The experimental results shows that the network incorporating the Anscombe transform and IT components has the best denoising performance. The introduction of the Anscombe transform plays a positive role in improving noise suppression performance. In photon-counting imaging, Poisson noise is more significant. This type of non-Gaussian noise makes the denoising process challenging. The Anscombe transform effectively alleviates the complexity of the noise distribution by converting Poisson noise into approximately Gaussian noise, reducing the modeling difficulty and improving noise suppression performance. This denoising structure has been proven to achieve better results in QIS denoising tasks [17]. If the Anscombe transform and its inverse process are not included, the neural network must autonomously learn this process through training, which is a relatively aggressive approach. Experimental results show that whether the IT component is included or not, the network configuration containing the Anscombe transform achieves slight improvements in PSNR, verifying the rationality and effectiveness of including the Anscombe transform in the network.

Secondly, the IT component also significantly improves the denoising performance of the network. To further enhance denoising performance, the IT component—which comprises FCL, ReLU, T, and residual learning—extracts various features across layers. These features include both linear and nonlinear structural information, as well as significant structural details derived from inter-image pixel relationships. Experimental results indicate that the exclusion of the IT component results in a significant drop in PSNR, demonstrating that the IT component plays a crucial role in enhancing the network’s denoising performance.

When both the Anscombe transform and IT component are introduced simultaneously, the network achieves optimal performance. This indicates that the Anscombe transform and IT components are complementary within the network: the former effectively mitigates the complex noise composition problem in QIS, providing higher-quality input data for subsequent network processing, while the latter further exploits image correlation information between networks.

### 4.2. Image Denoising Experimental Results

To comprehensively evaluate the denoising performance of the proposed QIS-SPFT, both quantitative and qualitative metrics were applied. For quantitative evaluation, the proposed QIS-SPFT was compared with three existing image reconstruction and denoising methods for QIS: the MLE method [15], the TD-BM3D method [17], QISNet [18] and DPIR [31]. PSNR was adopted to quantitatively assess the performance of QIS-SPFT. For grayscale synthetic QIS noisy images, the Set12 [29] and BSD68 [29] datasets were selected for testing, and four noise levels were set: Noise level 1 (*T* = 16, *K* = 4, $\sigma_{\mathrm{read}}$ = 0.2e-), Noise level 2 (*T* = 16, *K* = 4, $\sigma_{\mathrm{read}}$ = 0.4e-), Noise level 3 (*T* = 16, *K* = 2, $\sigma_{\mathrm{read}}$ = 0.2e-), Noise level 4 (*T* = 16, *K* = 1, $\sigma_{\mathrm{read}}$ = 0.4e-). The average PSNR results on the BSD68 dataset [29] are shown in Table 2.

The PSNR evaluation results for different noise levels on the Set12 dataset [29] are shown in Table 3.

To further illustrate the adaptability of the reconstruction method proposed in this paper across various scenarios, noisy images (*T* = 16, *K* = 3, $\sigma_{\mathrm{read}}$ = 0.2e-) were processed using three different datasets: Set12 [29], BSD68 [29] and Kodak24 [30]. The PSNR results were averaged for each dataset and the experimental result indicated that the proposed method outperformed the comparative methods. The experimental result is shown in Table 4.

Quantitative analysis results demonstrate that the proposed QIS-SPFT method exhibits significant performance advantages under various noise levels on the Set12 [29], BSD68 [29] and Kodak24 [30] datasets. Compared to existing methods MLE [15], TD-BM3D [17], QISNet [18] and DPIR [31], QIS-SPFT achieves higher PSNR values, proving its superiority in QIS image denoising tasks. The proposed method demonstrates excellent denoising results in both low-noise and high-noise scenarios, showing strong adaptability and robustness.

**Figure 6:**
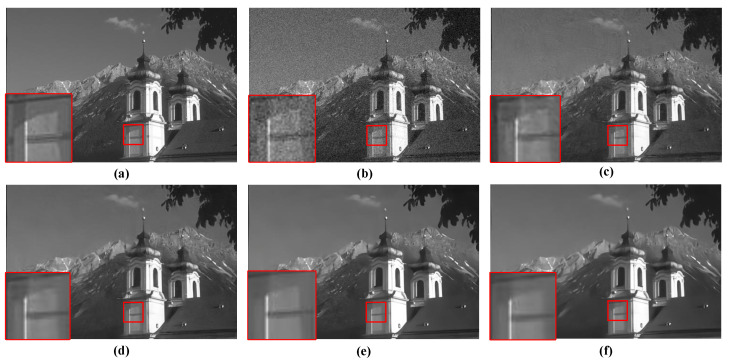
Comparison of denoising results of four methods for “castle” in BSD68 dataset: (**a**) Ground Truth, (**b**) MLE [15], (**c**) TD-BM3D [17], (**d**) QISNet [18], (**e**) DPIR [31], (**f**) Proposed.

**Figure 7:**
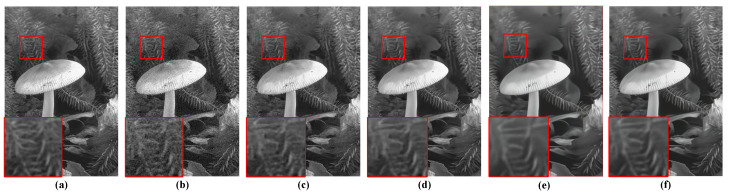
Comparison of denoising results of four methods for “mushroom” in BSD68 dataset: (**a**) Ground Truth, (**b**) MLE [15], (**c**) TD-BM3D [17], (**d**) QISNet [18], (**e**) DPIR [31], (**f**) Proposed.

**Figure 8:**
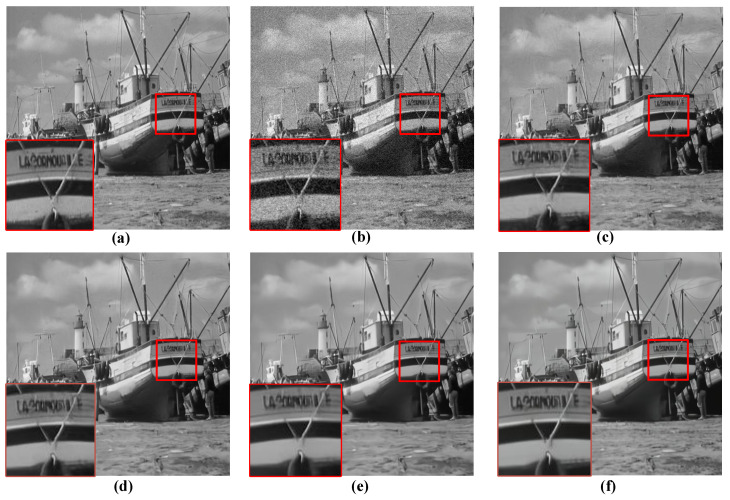
Comparison of denoising results of four methods for “boat” in Set12 dataset: (**a**) Ground Truth, (**b**) MLE [15], (**c**) TD-BM3D [17], (**d**) QISNet [18], (**e**) DPIR [31], (**f**) Proposed.

**Figure 9:**
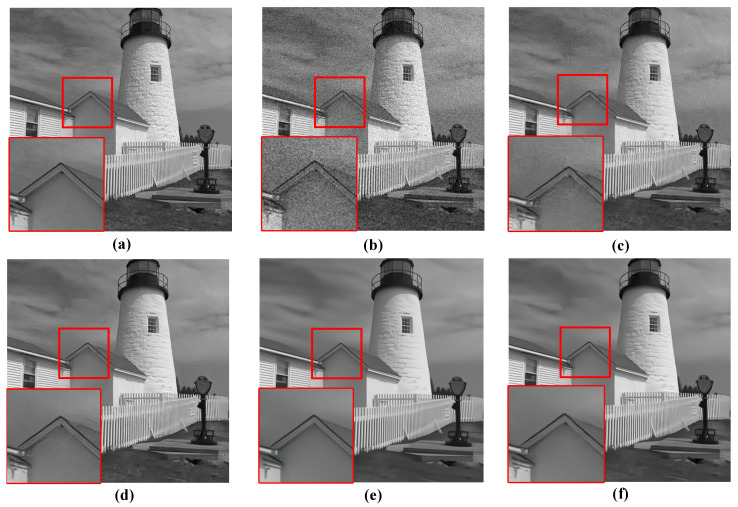
Comparison of denoising results of four methods for “house” in Kodak24 dataset: (**a**) Ground Truth, (**b**) MLE [15], (**c**) TD-BM3D [17], (**d**) QISNet [18], (**e**) DPIR [31], (**f**) Proposed.

**Figure 10:**
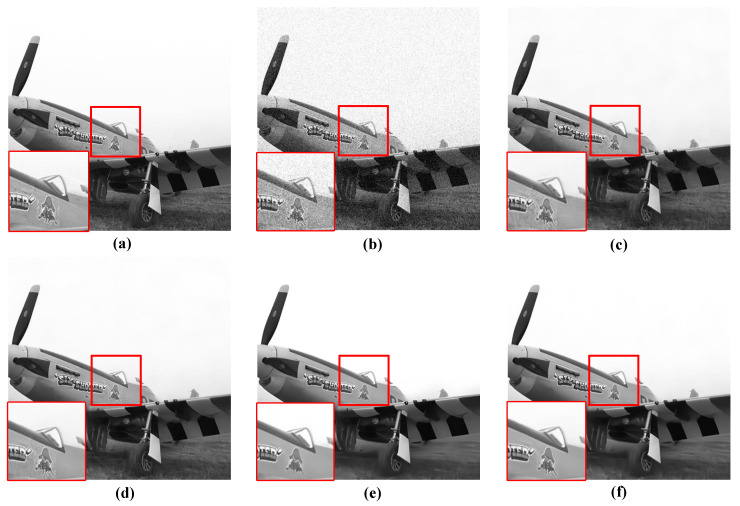
Comparison of denoising results of four methods for “airplane” in Kodak24 dataset: (**a**) Ground Truth, (**b**) MLE [15], (**c**) TD-BM3D [17], (**d**) QISNet [18], (**e**) DPIR [31], (**f**) Proposed.

For the qualitative evaluation of the denoising effectiveness of the proposed QIS-SPFT method, visual comparisons of reconstructed images were conducted. Specifically, regions of interest were selected to highlight key differences among different methods, as illustrated in Figure 6, Figure 7, Figure 8, Figure 9 and Figure 10. These figures provide a clear visual representation of how the proposed QIS-SPFT method enhances image clarity and effectively suppresses noise.

Compared with existing denoising techniques such as MLE [15], TD-BM3D [17], QISNet [18] and DPIR [31], QIS-SPFT demonstrates superior performance by preserving finer image details and improving the visibility of crucial regions. The results indicate that QIS-SPFT effectively reduces non-ideal noise while preserving the structural integrity and texture of the original images, which is essential for high-quality QIS image reconstruction. These findings further confirm that QIS-SPFT outperforms conventional denoising approaches and establishes itself as a robust method for enhancing imaging quality in QIS image reconstruction.

## 5. Discussion

- Loss of Image Texture: The proposed method is primarily designed to enhance image fidelity in the context of QIS reconstruction, where input signals are extremely noisy due to photon-limited conditions and sparse bit-plane encoding. In such scenarios, suppressing heavy noise and faithfully recovering the underlying image structure become the main objectives. As a result, our model is trained to minimize pixel-wise reconstruction error, which is well-reflected by metrics such as PSNR. However, this emphasis on fidelity can sometimes lead to a perceptual compromise. Specifically, the network exhibits a mild tendency toward over-smoothing, which may result in the suppression of some fine details, even when such details remain partially visible in the noisy input. This phenomenon stems from the inherent trade-off between aggressive noise suppression and texture preservation.To address this, our network architecture integrates modules for capturing local and global pixel correlations, which help preserve detailed structures to a certain extent. Nevertheless, the current design is still biased toward achieving higher fidelity scores, rather than optimizing for perceptual quality. To further improve the balance between these two aspects, future extensions of this work will consider introducing perceptual-oriented loss functions and incorporating structural attention mechanisms. These improvements are expected to enhance the retention of fine details and visual textures, particularly in important regions, while maintaining strong denoising performance under extreme noise conditions.
- Image Degradation: While the proposed QIS-SPFT framework demonstrates strong performance for photon shot noise suppression in QIS image reconstruction, it is specifically designed for binary bit-plane data under low-light conditions. Its generalization to other degradation types—such as motion blur [32] or haze [33]—has not been explored in this work. The blur degradation is a convolution of the Poisson process of imaging and require additional pre-processing or architectural adaptations. Equation (5) now can be described as:
  (18) $$P\left({\left[Y\right]}_{l}=y\right)=\frac{{\left[\gamma \cdot F\Theta_{\mathrm{tot}}\right]}_{l}^{y}e^{-{\left[\gamma \cdot F\Theta_{\mathrm{tot}}\right]}_{l}}}{y!},$$
  where F represents the blur kernel. The haze degradation follows the atmospheric scattering model. Future work may consider extending the framework to incorporate deblurring or dehazing modules to broaden its applicability.

## 6. Conclusions

This paper first introduces the shortcomings of current QIS noise reconstruction methods, then introduces the non-ideal imaging model of QIS imaging and proposes a noise suppression reconstruction method based on neural networks. The proposed neural network integrates convolutional neural networks and Transformer. Its architecture combines the Anscombe transformation with serial and parallel modules to enhance denoising performance and adaptability across various scenarios. Finally, the experiment demonstrates the rationality of the neural network architecture, showing that the reconstruction method proposed in this paper effectively suppresses photon shot noise in the QIS imaging process at a feasible and acceptable oversampling rate. This makes it an ideal solution for practical QIS imaging applications under extreme noise conditions. It achieves superior noise suppression compared to MLE [15], TD-BM3D [17], QISNet [18] and DPIR [31].

## Figures and Tables

**Table 1:** Network structure verification and comparison results.

Method	PSNR (dB)
QIS-SPFT without Anscombe, without IT	28.214
QIS-SPFT without Anscombe, with IT	28.253
QIS-SPFT with Anscombe, without IT	28.221
QIS-SPFT with Anscombe, with IT	28.269

**Table 2:** Average PSNR evaluation results under four different noise levels in the BSD68 dataset [29].

Methods	Noise Level 1	Noise Level 2	Noise Level 3	Noise Level 4
MLE [15]	20.32	19.98	19.65	13.84
TD-BM3D [17]	28.89	28.76	27.86	23.66
QISNet [18]	29.31	29.01	28.32	24.32
DPIR [31]	30.01	29.33	28.79	25.42
QIS-SPFT (proposed)	30.32	29.78	29.13	25.85

**Table 3:** PSNR evaluation results under four different noise levels in the Set12 dataset [29].

Images	Cameraman	House	Pepper	Starfish	Monarch	Parrot
Noise level 1						
MLE [15]	19.72	22.11	20.92	19.76	19.74	18.88
TD-BM3D [17]	29.21	33.32	31.44	30.31	29.34	29.04
QISNet [18]	29.74	33.77	31.91	30.89	29.83	29.64
DPIR [31]	30.43	34.42	32.63	31.52	30.62	30.31
QIS-SPFT (proposed)	30.77	34.75	32.80	31.95	30.91	30.75
Noise level 2						
MLE [15]	18.21	21.42	18.91	18.43	19.22	17.84
TD-BM3D [17]	28.77	31.64	30.21	29.02	29.88	28.44
QISNet [18]	29.33	32.12	30.84	29.54	30.34	29.01
DPIR [31]	29.72	33.53	31.23	30.30	30.77	29.33
QIS-SPFT (proposed)	30.07	33.92	31.71	30.65	31.02	29.84
Noise level 3						
MLE [15]	18.19	21.34	18.94	18.88	18.26	18.66
TD-BM3D [17]	27.22	31.24	28.34	27.21	27.84	27.34
QISNet [18]	28.41	31.86	28.91	27.86	28.52	27.88
DPIR [31]	29.66	32.91	30.64	29.15	29.72	28.81
QIS-SPFT (proposed)	29.90	33.24	30.89	29.46	30.13	29.16
Noise level 4						
MLE [15]	13.23	15.44	14.10	12.98	13.32	13.58
TD-BM3D [17]	23.91	25.71	23.32	23.11	23.97	24.23
QISNet [18]	24.32	26.15	23.91	23.74	24.51	24.72
DPIR [31]	25.88	27.02	26.41	24.97	26.24	26.58
QIS-SPFT (proposed)	26.10	28.34	26.85	25.35	26.62	26.86

**Table 4:** Average PSNR evaluation results under three different testing datasets.

Datasets	MLE [15]	TD-BM3D [17]	QISNet [18]	DPIR [31]	QIS-SPFT
**Set12** [29]	20.33	28.71	29.26	29.87	30.12
**BSD68** [29]	20.12	27.97	28.48	29.44	29.76
**Kodak24** [30]	20.85	29.24	30.12	30.68	31.13

## Data Availability

Data will be made available upon request.
